# Exercise serum promotes DNA damage repair and remodels gene expression in colon cancer cells

**DOI:** 10.1002/ijc.70271

**Published:** 2025-12-12

**Authors:** Samuel T. Orange, Emily Dodd, Sharanya Nath, Hannah Bowden, Alastair R. Jordan, Hannah Tweddle, Ann Hedley, Ifeoma Chukwuma, Ian Hickson, Sweta Sharma Saha

**Affiliations:** ^1^ School of Biomedical, Nutritional and Sport Sciences, Faculty of Medical Sciences, Newcastle University Newcastle upon Tyne UK; ^2^ Emles Bioventures London UK; ^3^ School of Medicine and Public Health, University of Wisconsin Madison Wisconsin USA; ^4^ School of Science, Technology and Health, York St John University York UK; ^5^ Translational and Clinical Research Institute, Faculty of Medical Sciences, Newcastle University Newcastle upon Tyne UK; ^6^ Bioinformatics Support Unit, Faculty of Medical Sciences, Newcastle University Newcastle upon Tyne UK; ^7^ Department of Biochemistry, Faculty of Biosciences University of Nigeria Enugu Nigeria; ^8^ School of Health & Life Sciences, Teesside University Middlesbrough UK

**Keywords:** colon cancer, DNA damage, DNA repair, exercise, radiation

## Abstract

Exercise protects against colon cancer progression, but the underlying biological mechanisms remain unclear. One proposed mechanism is the release of bioactive molecules into the systemic circulation during exercise, which may act directly on tumour cells to suppress DNA damage, inhibit proliferation, and preserve genomic stability. Here, we profiled the serum proteomic response to acute exercise and evaluated the effects of exercise‐conditioned human serum on DNA damage kinetics and transcriptomic signatures in colon cancer cells. Blood samples were collected from 30 overweight/obese adults before and immediately after a maximal incremental cycling test. LoVo cells were exposed to pre‐ or post‐exercise serum, treated with 2 Gy irradiation, and assessed for γ‐H2AX foci over 24 h. Acute exercise increased the relative abundance of 13 proteins in serum (*p* < 0.05), including interleukin‐6 (IL‐6) and its soluble receptor IL‐6R, reflecting systemic activation of acute‐phase immune and vascular signalling. Compared to pre‐exercise serum, post‐exercise serum significantly reduced γ‐H2AX foci in LoVo cells at 6 h (*p* = 0.010) and decreased the area under the curve (*p* = 0.014), indicating accelerated DNA repair. Post‐exercise serum also increased expression of the DNA repair gene *PNKP*, with and without irradiation (*p* = 0.007 and *p* = 0.029, respectively). Transcriptomic analysis revealed upregulation of mitochondrial energy metabolism and downregulation of cell cycle and proteasome‐related pathways. These findings suggest that acute exercise elicits systemic responses that enhance DNA repair and shift colon cancer cells towards a less proliferative transcriptomic state under sublethal genotoxic stress, offering a potential mechanistic explanation for the protective effects of exercise against colorectal carcinogenesis.

## What's New?

Exercise releases bioactive molecules into the bloodstream that can directly slow cancer cell growth. In colon cancer, this may help limit disease progression. Here, using colon cancer cells, the authors investigated the effects of exercise‐conditioned human serum on DNA repair mechanisms. Notably, acute exercise in humans elicited systemic responses, altering the circulating proteome. Resulting effects included enhanced DNA repair and activation of gene expression signatures consistent with increased mitochondrial metabolism and reduced proliferation in colon cancer cells. The findings indicate that acute exercise could suppress colorectal carcinogenesis via mechanisms involved in the regulation of DNA repair.

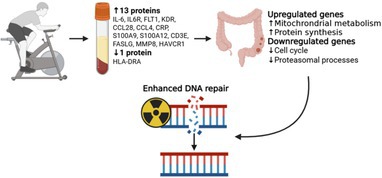

**Abbreviations**
AMPK: 5' AMP‐activated protein kinase
BMI: body mass index
BSA: bovine serum albumin
CCL4: C‐C motif chemokine ligand 4
CCL28: C‐C motif chemokine ligand 28
CD3E: CD3 epsilon chain
CRP: C‐reactive protein
DAPI: 4',6‐diamidino‐2‐phenylindole
DMEM/F12: Dulbecco's Modified Eagle Medium/Nutrient Mixture F‐12
DSB: double‐strand break
FASLG: Fas ligand
FCS: fetal calf serum
FDR: false discovery rate
FLT1: Fms‐related tyrosine kinase 1 (vascular endothelial growth factor receptor 1)
GO: Gene Ontology
Gp130: Glycoprotein 130
Gy: Gray
HAVCR1: hepatitis A virus cellular receptor 1
HLA‐DRA: Human leukocyte antigen DR alpha
IL‐6: interleukin 6
IL6R: interleukin 6 receptor
KDR: Kinase insert domain receptor (vascular endothelial growth factor receptor 2)
log₂FC: log₂ fold change
MMP8: matrix metalloproteinase 8
NHEJ: non‐homologous end joining
OXPHOS: oxidative phosphorylation
PBS: phosphate‐buffered saline
PCA: principal component analysis
PGC‐1α: Peroxisome proliferator‐activated receptor gamma coactivator 1‐alpha
PNKP: polynucleotide kinase 3′‐phosphatase
PPARγ: Peroxisome proliferator‐activated receptor gamma
qPCR: quantitative polymerase chain reaction
RER: respiratory exchange ratio
RNA‐seq: RNA sequencing
RPE: rating of perceived exertion
S100A9: S100 calcium‐binding protein A9
S100A12: S100 calcium‐binding protein A12

## 1 INTRODUCTION

Regular exercise suppresses colon cancer progression.^1^, ^2^, ^3^ Supervised exercise following adjuvant chemotherapy improves disease‐free survival,^4^ and voluntary exercise reduces chemically induced intestinal tumours in preclinical models.^1^, ^2^ Putative biological mechanisms include enhanced immunosurveillance and reduced chronic inflammation and insulin resistance.^5^ However, targeting these pathways has shown limited efficacy in improving colon cancer outcomes,^6^, ^7^, ^8^ and both chronic inflammation and insulin resistance appear to be regulated primarily by adiposity rather than exercise per se.^9^, ^10^ Exercise improves colon cancer outcomes without changing adiposity,^4^ suggesting that additional mechanisms may be involved.

Emerging evidence demonstrates that bioactive molecules (e.g., proteins, nucleic acids, metabolites) released into the systemic circulation during exercise can act directly on cancer cells to inhibit tumour progression.^5^, ^11^ These exercise‐induced molecules, collectively known as ‘exerkines’, exert systemic effects on recipient cells through endocrine‐like signalling. We previously showed that exposing colon cancer cells to human serum sampled immediately post‐exercise reduced cell proliferation by 6% compared to control (non‐exercise) serum.^12^ This was accompanied by a 25% decrease in γ‐H2AX expression, a marker of DNA double‐strand breaks (DSBs). Acute exercise also elevated serum interleukin (IL‐6)—an established exerkine—and recombinant IL‐6 reduced cell proliferation and DNA damage in a dose‐dependent manner, mimicking the effects of exercise serum.^12^

Given that cancer cell lines rapidly attain new genetic variants in culture that accelerate proliferation,^13^ exercise‐induced reductions in DNA damage may limit colon cancer cell proliferation by promoting genomic stability.^12^ This aligns with the oncogene‐induced DNA damage model for cancer development, which proposes that persistent DNA DSBs drive cancer progression by increasing the propensity of acquiring genetic mutations that enhance cell proliferation and metastatic potential.^14^

Defining how exercise influences DNA damage repair in colon cancer may yield important insights into its protective effects. A deeper mechanistic understanding could help identify biomarkers for risk stratification, inform exercise‐mimetic drug development, or establish surrogate endpoints for trials. Here, we: (i) profiled the circulating proteomic response to acute exercise in humans, (ii) investigated the effects of exercise‐conditioned human serum on DNA damage kinetics in irradiated colon cancer cells, and (iii) examined associated transcriptomic changes to explore potential underlying mechanisms.

## 2 METHODS

### 2.1 Study design

LoVo colon cancer cells were exposed to pre‐ or post‐exercise human serum, treated with 2 Gray (Gy) irradiation to induce DNA DSBs, and assessed for nuclear γ‐H2AX foci formation over 24 h. Serum samples were generated from 30 participants who performed an acute exercise bout, with blood samples collected pre‐ and immediately post‐exercise. Prior to the exercise bout, participants fasted overnight (>10 h), avoided structured exercise (≥48 h), alcohol (≥24 h) and caffeine (≥12 h), and arrived hydrated. All experiments used individual (non‐pooled) serum samples.

### 2.2 Participants

Thirty apparently healthy adults aged 50–78 years completed the exercise trial. Inclusion criteria were age ≥50 years and being classified as overweight or obese based on a body mass index (BMI) of 25–39.9 kg/m^2^. Exclusion criteria were: (i) pre‐existing cardiovascular, metabolic, or renal disease; (ii) fasting blood glucose ≥7 mmol/L; (iii) hypertension (≥160/≥90 mm Hg); (iv) previous stroke or treatment for malignancy; (v) respiratory disease with peak expiratory flow <300 L/min; or (vi) any physical condition that could be exacerbated by exercise, as determined by the research team. Participant socio‐demographics, medical history, BMI, waist circumference, body composition (Seca mBCA 515, Hamburg, Germany), peak expiratory flow, and fasting blood glucose (Biosen C‐Line, EKF Diagnostics, UK) were recorded (Table 1).

**TABLE 1:** Participant characteristics.

	Total (*n* = 30)	Male (*n* = 18)	Female (*n* = 12)
Age (years)	64.1 ± 8.4	65.3 ± 7.1	64.3 ± 7.5
Ethnicity			
White British	29 (97%)	17 (94%)	12 (100%)
Multiple ethnic groups	1 (3%)	1 (6%)	0 (0%)
V̇O_2_peak (ml.kg^−1^.min^−1^)	27.2 ± 6.7	29.7 ± 7.4	23.5 ± 2.9
Body mass (kg)	85.7 ± 11.0	90.3 ± 8.4	78.8 ± 11.0
BMI (kg/m^2^)	29.6 ± 2.9	29.5 ± 2.2	29.7 ± 3.8
Waist circumference (cm)	98.9 ± 8.8	102 ± 6.7	93.2 ± 9.3
Body fat (%)^a^	35.5 ± 6.9	30.5 ± 3.2	43.1 ± 2.2
Fat‐free mass (%)^a^	64.5 ± 6.8	69.5 ± 3.2	57.1 ± 2.0
Skeletal muscle mass (%)^a^	29.3 ± 8.9	34.4 ± 2.3	22.6 ± 10.0
Fasting BG (mmol/L)	4.7 ± 0.8	4.7 ± 0.9	4.8 ± 0.7
Peak expiratory flow (L/min)	462 ± 120	536 ± 91.9	350 ± 50.8

*Note*: Data presented as mean ± SD or number (%).

Abbreviations: BG, blood glucose; BMI, body mass index; V̇O_2_peak, peak oxygen consumption.

^a^ *n* = 15 due to bioelectrical impedance analysis not being available for all participants.

### 2.3 Sample size justification

Our primary outcome was the difference in γ‐H2AX foci after exposure to pre‐ versus post‐exercise serum. In our prior study, post‐exercise serum reduced γ‐H2AX expression in colon cancer cells by 25 ± 47% (Cohen's *d*_*z*_ = 0.53).^12^ A sample size of *n* = 30 participants provides 80% power to detect this effect (matched pairs) with a two‐tailed α = 0.05.

### 2.4 Exercise trial

After fasting overnight, participants performed a maximal incremental exercise test between 08:00 and 10:00 on an electronically braked cycle ergometer (VIAsprint 200P, Ergoline GmbH, Germany). After a 3 min warm‐up against no resistance, work rate increased by 15–25 W·min^−1^ to reach volitional exhaustion in 10–12 min. Cadence was maintained at 60–75 rev·min^−1^; volitional exhaustion was defined as a ≥10 rev·min^−1^ drop in cadence for five consecutive seconds despite strong verbal encouragement. Breath‐by‐breath data (Vyntus CPX, Vyaire Medical Inc., Chicago, IL), heart rate (Polar H10, Polar Electro, Finland), blood pressure, and rating of perceived exertion (RPE, 6–20 Borg scale) were recorded throughout. All but one participant achieved a peak respiratory exchange ratio (RER) of >1.10 (mean RER = 1.21 ± 0.10), confirming maximal effort.

### 2.5 Blood sampling

Venous blood samples (≈30 mL) were drawn from an antecubital vein before and within 1 min of exercise cessation. Samples were collected in 10 mL Vacutainer serum tubes (BD, New Jersey), inverted 5–10 times, allowed to clot at room temperature for 30 min, centrifuged (1500 g, 15 min), aliquoted (≈0.5 mL), and cryopreserved at −80°C.

### 2.6 Cell line

The LoVo cell line (RRID:CVCL_0399) harbours *APC* and *KRAS* mutations but is wildtype for *TP53*,^15^ a genotype typically associated with early stages of colon cancer development.^16^ Although originally derived from a metastatic lesion, LoVo cells retain key molecular features of early‐stage disease and display microsatellite instability,^15^ a genomic instability phenotype present in a subset of early tumours. These characteristics make the LoVo cell line a suitable model to investigate the effects of exercise on DNA repair and gene expression signatures relevant to early‐stage colon tumorigenesis.^12^ Cells were purchased from Sigma‐Aldrich (Dorset, UK) and cultured in DMEM/F12 (1:1) with 10% foetal calf serum, 1% glutamine, and 1% penicillin–streptomycin. For experiments, cells were passaged 4–8 times at ≈70–80% confluence and maintained at 37°C in a humidified atmosphere of 5% CO_2_ and ~21% O_2_. All cell lines were authenticated using short tandem repeat (STR) profiling within the last 3 years. All experiments were performed with mycoplasma‐free cells.

### 2.7 DNA damage repair

To assess DNA repair, nuclear γ‐H2AX foci were quantified by immunofluorescence. LoVo cells were seeded at 5 × 10^4^ cells·well^−1^ in a 6‐well plate for 48 h, washed with phosphate‐buffered saline (PBS), serum‐starved for 2 h (0.2% bovine serum albumin [BSA] in DMEM), then treated with media containing: (i) 10% FCS; (ii) 10% pre‐exercise serum; or (iii) 10% post‐exercise serum. Paired pre‐ and post‐exercise samples from the same participant (*n* = 30) were used on the same 6‐well plate to minimise interplate variability.^12^ After 1 h serum stimulation, cells were irradiated with 2 Gy of x‐ray radiation (0.8 min) and fixed at 5 min, 1 h, 6 h, and 24 h. Cells were permeabilised with 0.2% Triton X‐100 in PBS (PBS‐Triton), blocked with PBS containing 2% BSA, 10% milk, and 10% goat serum, then incubated for 1 h with mouse anti‐phospho‐H2AX antibody (1:1000, EMD Millipore) followed by Alexa Fluor 647‐conjugated goat anti‐mouse secondary antibody (1:1000, Abcam) in PBS‐Triton with 2% BSA. Nuclei were counterstained with 4′,6‐diamidino‐2‐phenylindole (DAPI, 1:1000). Images were captured using a Leica DM6 microscope (40× objective; LasX 3.4.2). γ‐H2AX foci were quantified in ≥100 cells per coverslip using ImageJ (FIJI). Background counts from unirradiated controls were subtracted, and values were normalised to the 5 min post‐irradiation timepoint. The area under the curve (AUC) for γ‐H2AX over the 24 h was calculated using the trapezoidal rule.

### 2.8 RNA sequencing

LoVo cells were treated with DMEM containing 10% pre‐ and post‐exercise serum from a subsample of 12 participants (see Table S1). Cells were exposed to individual participant serum for 6 h, then harvested via trypsinisation, washed with PBS, and snap‐frozen in liquid nitrogen. RNA extraction, library preparation, and sequencing were performed by Novogene (Cambridge, UK) using standard protocols. RNA quality was assessed using Nanodrop, Qubit, and Bioanalyzer instruments. Libraries were sequenced on an Illumina NovaSeq 6000, generating ~25 million paired‐end reads/sample. Reads were aligned to the human genome (GRCh38), and transcript abundance was quantified. Differential expression analysis was performed in R using DESeq2 (version 1.38.3), with significance set at false discovery rate (FDR) <0.05 (Benjamini‐Hochberg corrected). Gene Ontology (GO) enrichment analysis was conducted using DAVID (https://davidbioinformatics.nih.gov/). Principal component analysis (PCA) was conducted on the top differentially expressed genes. The sequencing coverage and quality statistics for each sample are summarized in Table S2.

### 2.9 Real‐time quantitative PCR

RNA (1 μg) extracted from LoVo cells was reverse‐transcribed using the High‐Capacity cDNA Reverse Transcription Kit with RNase inhibitor (Thermo Fisher, Cat #4374966) in 20 μL reactions with random hexamers. The reaction was incubated at 25°C for 10 min, 37°C for 2 h, and 85°C for enzyme inactivation. LoVo cells stimulated with paired pre‐ and post‐exercise serum (±2 Gy irradiation) were prepared for 11 participants, using the same subsample as the RNA sequencing (RNA‐seq) analysis, with one participant excluded due to insufficient serum. Quantitative polymerase chain reaction (qPCR) was performed using the Bio‐Rad CFX Maestro system with GAPDH as the reference gene. Gene expression was analysed using the ΔCt method across technical replicates. Primer sequences are provided in Table S3.

### 2.10 Serum proteomic profiling

Multiplex profiling of serum inflammatory proteins was performed using the NULISAseq™ Inflammation Panel AQ (Alamar Biosciences, Fremont, CA, USA), a high‐sensitivity nucleic acid‐linked immunoassay enabling the relative quantification of ~249 targets and absolute quantification of ~157 proteins.^17^ Serum samples were thawed and centrifuged at 2200 g for 10 min. A 25 μL aliquot of supernatant was assayed on the automated Alamar ARGO HT system (Alamar Biosciences) at Altheome Limited laboratories (London, UK). Following immunocomplex formation with paired oligo‐conjugated antibodies, sequential capture (oligo‐dT bead capture, release, streptavidin bead recapture, and ligation) generated DNA reporter libraries containing target‐ and sample‐specific molecular identifiers, which were PCR‐amplified, purified, and sequenced on the Element AVITI system. The assay was performed on paired serum samples from 12 participants (Table S1). Relative quantification data normalized to standard controls were used for analysis. The median intra‐plate coefficient of variation across all analytes was 5.9%.

### 2.11 Statistical analysis

A linear mixed‐effects model was used to compare γ‐H2AX AUC between pre‐ and post‐exercise serum conditions, with condition as a fixed effect and random slopes for participants to account for repeated measures. A further mixed‐effects model was used to compare γ‐H2AX foci at 1, 6, and 24 h post‐irradiation, with condition, timepoint, and their interaction as fixed effects, and random slopes for participants. Pairwise comparisons at each timepoint were obtained using estimated marginal means. Normality of residuals was assessed by visual inspection of histograms and Q–Q plots. Relative changes in gene expression (ΔCt values) were analysed using paired t‐tests. Serum protein differences were analysed using paired t‐tests without correction for multiple comparisons, as the serum proteomic profiling was considered exploratory. Statistical significance was defined as *p* < 0.05. Analyses were performed in R (v4.4.3). Data and code are available on Open Science Framework (https://osf.io/2adyh/).

## 3 RESULTS

### 3.1 Exercise serum promotes DNA damage repair in colon cancer cells

**FIGURE 1:**
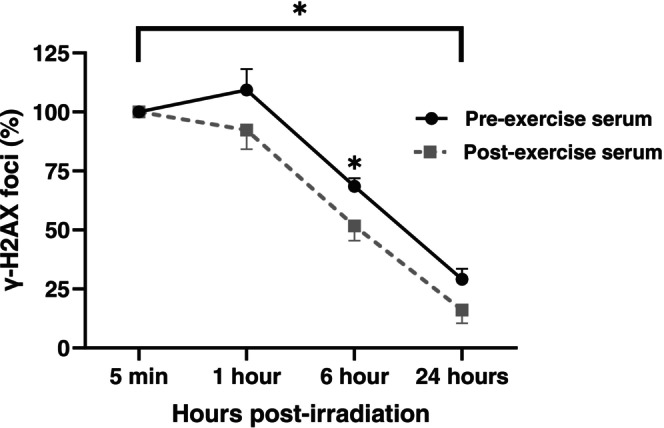
Exercise serum accelerates DNA damage repair in irradiated colon cancer cells. LoVo cells were irradiated (2 Gy) following 1 h stimulation with human serum collected before or immediately after acute exercise. γ‐H2AX foci were quantified at 5 min, 1 h, 6 h, and 24 h post‐irradiation as a marker of DNA double‐strand breaks. Data are expressed as a percentage of γ‐H2AX foci relative to the 5‐min time point. Post‐exercise serum significantly reduced γ‐H2AX foci area under the curve (AUC) and levels at 6 h post‐irradiation, indicating accelerated DNA damage repair. *p < 0.05.

To investigate whether exercise serum modulates DNA repair, we stimulated LoVo cells with medium containing 10% human serum collected pre‐ and post‐exercise. Following 1 h serum exposure, cells were treated with 2 Gy x‐ray irradiation, and γ‐H2AX foci kinetics were assessed over 24 h. Compared to pre‐exercise serum, post‐exercise serum significantly reduced γ‐H2AX foci AUC (−356 arbitrary units, 95% CI: −630 to −83.0 arbitrary units, *p* = 0.014), indicating an exercise‐induced acceleration of DNA damage repair (Figure 1). Exercise serum also reduced γ‐H2AX foci at 6 h post‐irradiation (−16.8%, 95% CI: −29.5 to −4.0%, *p* = 0.010). There was no evidence of an effect of exercise‐conditioned serum on γ‐H2AX foci levels at 1 h (−17.1%, 95% CI: −41.3 to 7.2%, *p* = 0.17) or 24 h (−13.1%, 95% CI: −28.0 to 1.8%, *p* = 0.085) post‐irradiation.

### 3.2 Exercise serum induces transcriptomic signatures of bioenergetic activation and proteostasis

**FIGURE 2:**
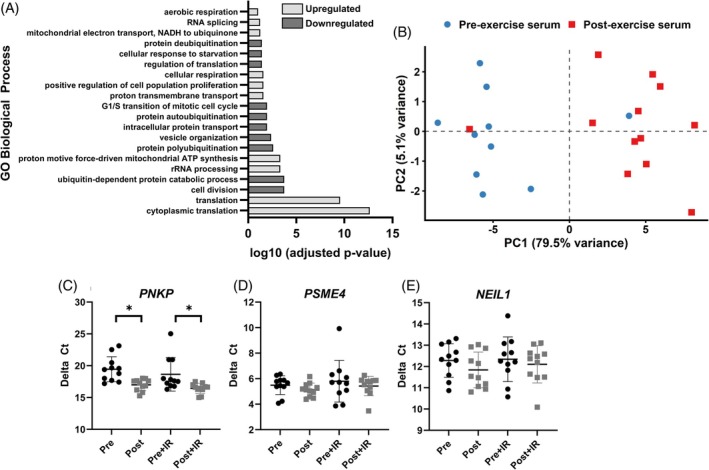
Exercise serum induces transcriptional signatures of enhanced bioenergetics and proteostasis and upregulates the DNA repair enzyme *PNKP* in colon cancer cells. (A) Gene Ontology (GO) enrichment analysis of genes upregulated in LoVo cells stimulated with post‐exercise serum revealed over‐representation of biological processes related to protein synthesis and mitochondrial energy metabolism. (B) Principal component analysis (PCA) of the top differentially expressed genes demonstrated clear separation between LoVo cells stimulated with pre‐ vs. post‐exercise serum, indicating distinct transcriptional profiles. (C) qPCR validation confirmed that post‐exercise serum significantly upregulated *PNKP*, a key gene involved in DNA repair via base excision repair. (D, E) qPCR did not confirm differential expression of *PSME4* or *NEIL1*. *p < 0.05.

To explore potential mechanisms underlying enhanced DNA repair, we performed RNA‐seq on LoVo cells stimulated with pre‐ or post‐exercise serum. Post‐exercise serum altered the expression of 1364 genes compared with pre‐exercise serum (FDR <0.05), including 627 that were upregulated and 737 downregulated. Log₂ fold changes (log₂FC) ranged from −0.54 to 1.25 (median = −0.11). GO analysis of upregulated genes revealed enrichment of biological processes related to protein synthesis (e.g., cytoplasmic translation, rRNA processing) and mitochondrial energy metabolism (e.g., oxidative phosphorylation, cellular respiration) (Figure 2A). Downregulated genes were enriched in pathways linked to cell cycle progression (e.g., cell division, G1/S transition of mitotic cell cycle) and proteasomal processes (e.g., protein polyubiquitination, deubiquitination). PCA of the top differentially expressed genes confirmed clear separation between pre‐ and post‐exercise conditions (Figure 2B).

### 3.3 Exercise serum upregulates expression of DNA repair gene *PNKP*

To assess exercise effects on DNA repair gene expression, we selected *PNKP*, *PSME4*, and *NEIL1* for validation by qPCR based on transcriptomic changes and roles in DNA repair. *PNKP*, which encodes a DNA kinase‐phosphatase essential for base excision repair, was significantly upregulated in the RNA‐seq dataset (log₂FC = 0.15, FDR = 0.035). qPCR confirmed increased *PNKP* expression following post‐exercise serum stimulation: 1.9‐fold (non‐irradiated, *p* = 0.007) and 4.5‐fold (irradiated, *p* = 0.029) relative to pre‐exercise serum (Figure 2C). qPCR did not reveal any significant changes in the expression of *PSME4* or *NEIL1* following post‐exercise serum stimulation (*p* > 0.05, Figure 2D,E).

### 3.4 Exercise alters circulating inflammatory and vascular proteins

**FIGURE 3:**
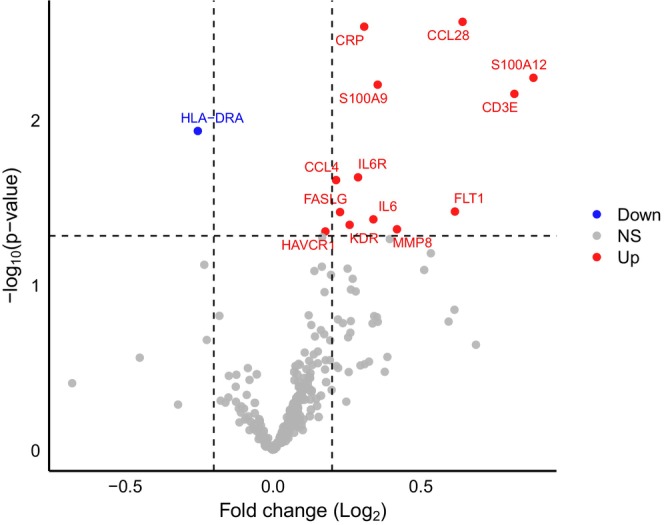
Exercise alters circulating inflammatory and vascular proteins. Volcano plot showing changes in the relative abundance of serum proteins following an acute bout of exercise. Acute exercise significantly (*p* < 0.05) increased the abundance of 13 proteins, including interleukin‐6 (IL‐6), interleukin‐6 receptor (IL6R), Fms‐related tyrosine kinase 1 (FLT1), kinase insert domain receptor (KDR), C‐C motif chemokine ligand 28 (CCL28), C‐C motif chemokine ligand 4 (CCL4), C‐reactive protein (CRP), S100 calcium‐binding protein A9 (S100A9), S100 calcium‐binding protein A12 (S100A12), CD3 epsilon chain (CD3E), Fas ligand (FASLG), matrix metalloproteinase‐8 (MMP8), and hepatitis A virus cellular receptor 1 (HAVCR1). These changes are consistent with systemic activation of acute‐phase immune, vascular, and metabolic signalling. Serum human leukocyte antigen DR alpha (HLA‐DRA) was significantly reduced post‐exercise, suggesting transient downregulation of antigen presentation and adaptive immune activity. NS, non‐significant.

Finally, we profiled the serum proteome to elucidate the systemic factors potentially responsible for the observed effects of exercise serum on DNA repair and gene expression in colon cancer cells. Acute exercise increased the relative abundance of 13 serum proteins (*p* < 0.05), including IL‐6, IL6R, FLT1, KDR, CCL28, CCL4, CRP, S100A9, S100A12, CD3E, FASLG, MMP8, and HAVCR1 (Figure 3), reflecting systemic activation of acute‐phase immune, vascular, and metabolic signalling. Exercise also resulted in a significant decrease in serum HLA‐DRA (*p* < 0.05), consistent with a transient downregulation of antigen presentation and adaptive immune activity. Absolute protein quantification data are provided on Open Science Framework (https://osf.io/2adyh/).

## 4 DISCUSSION

This study demonstrates that exercise‐conditioned human serum promotes the mechanisms responsible for DSB repair in colon cancer cells and induces transcriptomic signatures associated with enhanced bioenergetic activation and reduced cell cycle progression. These findings lend support to the hypothesis that systemic responses to acute exercise may suppress colon cancer progression, in part, by promoting DNA repair and shifting cells towards a less proliferative state under sublethal genotoxic stress.

Exposing colon cancer cells to post‐exercise serum reduced γ‐H2AX foci following treatment with 2 Gy ionising radiation. This sublethal dose of radiation (2 Gy) induces DSBs and point mutations, including base substitutions, frameshifts, and small deletions, through misrepair mechanisms.^18^, ^19^ Persistent sublethal genotoxic stress, particularly under conditions of error‐prone repair, can promote the survival and expansion of genetically unstable clones.^14^ Enhancing DSB repair through exercise may therefore preserve genomic stability and limit clonal evolution. Although one proliferation‐related pathway was modestly upregulated (positive regulation of cell population proliferation, Figure 2A), the overall transcriptional profile—marked by downregulation of cell cycle processes (cell division; G1/S transition of mitotic cell cycle) and proteasome/ubiquitin pathways (ubiquitin‐dependent protein catabolic process; protein polyubiquitination; protein autoubiquitination; protein deubiquitination)—indicates a shift away from active proliferation, consistent with our prior findings.^11^ However, the relevance of enhanced DNA repair in the context of cytotoxic therapies, which aim to induce irreparable DNA damage, remains uncertain and warrants further investigation.

Post‐exercise serum upregulated mitochondrial pathways in LoVo cells, including oxidative phosphorylation (OXPHOS), electron transport, and aerobic respiration. Mitochondrial dysfunction contributes to colon cancer progression by increasing oxidative stress, impairing redox balance, and promoting genomic instability.^20^ The observed exercise‐induced enrichment of mitochondrial pathways may suppress pro‐tumour metabolic processes by enhancing OXPHOS and redox control, potentially reversing the glycolytic phenotype typical of many colorectal cancers.^21^ Indeed, enhancing mitochondrial biogenesis through AMPK‐PGC‐1α signalling—a pathway known to be activated by exercise^22^—can restore OXPHOS and inhibit colon cancer progression.^23^ In vivo, increased OXPHOS may reduce tumour hypoxia, creating a microenvironment more conducive to vascular normalisation and increased tumour perfusion.^24^ Several of the serum proteins that increased post‐exercise, including FLT1, KDR, IL‐6, and MMP8, are involved in angiogenic and vascular remodelling pathways that could further these effects. Additionally, improved mitochondrial function may support more effective DNA repair, particularly energy‐dependent single‐strand break repair and non‐homologous end joining (NHEJ), by maintaining cellular ATP levels and redox homeostasis essential for repair enzyme activity.

Acute exercise increased the relative abundance of 13 serum proteins, including IL‐6 and its soluble receptor IL‐6R. IL‐6 promotes hepatic synthesis of acute‐phase proteins and mobilises innate immune cells, such as neutrophils and lymphocytes, which can shed membrane‐bound IL‐6Rα to generate soluble IL‐6R.^5^ The concurrent rise in serum IL‐6 and IL‐6R indicates activation of IL‐6 trans‐signalling via gp130 dimerisation on target cells, a pathway known to enhance mitochondrial energy metabolism through AMPK–PGC‐1α and PPARγ signalling.^25^, ^26^, ^27^ These downstream effects may partly underlie the observed upregulation of mitochondrial pathways and improved DNA repair capacity in LoVo cells. Consistent with this, we previously showed that recombinant IL‐6 reduces DNA damage and proliferation in LoVo cells in a dose‐dependent manner.^12^ The gene expression changes identified here may also reflect redox‐sensitive signalling driven by transient increases in reactive oxygen species during acute exercise, in line with the rise in serum proteins linked to oxidative stress, such as IL‐6, CRP, S100A9, and S100A12.

*PNKP* gene expression was upregulated in colon cancer cells stimulated with post‐exercise serum, both with and without irradiation (Figure 2C). *PNKP* encodes a critical enzyme involved in the repair of DNA single‐ and double‐strand breaks via base excision repair and NHEJ pathways, respectively. Although clinical transcriptomic data show no consistent differences in *PNKP* expression between tumour grades,^28^ its function may still be modulated by post‐transcriptional regulation or by features of the tumour microenvironment, such as hypoxia and oxidative stress.^29^, ^30^ In preclinical models, *PNKP* inhibition sensitises cells to lethal radiation and induces synthetic lethality in PTEN‐deficient cancers.^31^ In contrast, the exercise‐induced upregulation of *PNKP* observed here may reflect an adaptive transcriptional response that enhances DNA repair and genomic stability under sublethal genotoxic stress, such as that arising from oxidative or replicative damage within the tumour microenvironment or during early carcinogenesis.^32^, ^33^ Further research should determine whether increased *PNKP* expression translates to functional effects on tumour behaviour in vivo.

Exercise is increasingly recognised as an adjunct therapy in cancer care. A recent trial found that a 3‐year supervised exercise programme following chemotherapy improved disease‐free survival in patients with colon cancer, primarily through reduced recurrence and new tumours.^4^ These effects occurred without significant changes in body weight, suggesting that adiposity‐related mechanisms are unlikely to explain the observed effects. Based on our findings, we hypothesise that regulation of DNA repair may be one mechanism through which exercise improves colon cancer outcomes.

This study has some limitations. We used a single two‐dimensional cell culture model, which does not capture the full complexity of tumour heterogeneity or the microenvironment. However, genomic instability driven by oncogene‐induced DNA damage is a hallmark of many cancers.^14^ Therefore, the observed effects of exercise‐conditioned serum on DNA repair are likely to be relevant to other epithelial malignancies, including other colorectal cancers, in which persistent genotoxic stress contributes to tumour development. The acute exercise trial was brief (~10–12 min) but involved maximal effort, which may not be feasible or appropriate for some individuals with cancer, particularly during active treatment or for those with co‐morbidities. As the systemic responses to exercise are at least partly intensity‐dependent,^34^ it remains unclear whether lower‐intensity exercise would elicit a similar serum proteomic response or biological effect on cancer cells. Additionally, the serum samples were collected from apparently healthy, overweight and obese adults rather than individuals with cancer. This decision reflects the design of our experimental model, which aimed to isolate and apply the systemic molecular response to acute exercise (i.e., the exerkine response) to colon cancer cells in vitro. Evidence suggests that people with obesity and people with cancer exhibit broadly similar acute molecular responses to exercise, including rises in the pleiotropic exerkine IL‐6,^12^, ^35^ supporting the choice to recruit healthy volunteers in this experimental context.

In conclusion, acute exercise altered the circulating proteome, enhanced DNA damage repair and expression of the DNA repair gene *PNKP* in colon cancer cells, and induced gene expression signatures indicative of improved mitochondrial metabolism and reduced proliferation. These findings identify the regulation of DNA repair as a potential mechanistic link between acute exercise and the suppression of colorectal carcinogenesis, strengthening the rationale for integrating exercise as an adjunct to standard care in the treatment of colon cancer.

## AUTHOR CONTRIBUTIONS

**Samuel T. Orange:** Conceptualization; methodology; funding acquisition; visualization; writing – original draft; project administration; supervision; investigation; formal analysis. **Emily Dodd:** Investigation; writing – review and editing; data curation. **Sharanya Nath:** Data curation; investigation; writing – review and editing. **Hannah Bowden:** Data curation; investigation; writing – review and editing. **Alastair R. Jordan:** Investigation; data curation; resources; writing – review and editing. **Hannah Tweddle:** Investigation; writing – review and editing. **Ann Hedley:** Formal analysis; data curation; resources; writing – review and editing. **Ifeoma Chukwuma:** Writing – review and editing; validation; investigation. **Ian Hickson:** Conceptualization; funding acquisition; methodology; supervision; writing – review and editing. **Sweta Sharma Saha:** Conceptualization; funding acquisition; project administration; writing – review and editing; supervision; methodology; investigation.

## FUNDING INFORMATION

This research received funding from the Wellcome Trust Institutional Strategic Support Fund (ISSF) Small Grants Scheme.

## CONFLICT OF INTEREST STATEMENT

The authors declare no conflicts of interest.

## ETHICS STATEMENT

The study was approved by the Faculty of Medical Sciences Research Ethics Committee, part of Newcastle University's Research Ethics Committee (ref: 02219). Written informed consent was obtained from all participants prior to data collection.

## Supporting information


**DATA S1.** Supporting Information.

## Data Availability

All data and code are available on the Open Science Framework project page (https://osf.io/2adyh/). Further information is available from the corresponding author upon request.
