# Supplementary material for: Exercise serum promotes DNA damage repair and remodels gene expression in colon cancer cells
Source: Int J Cancer. 2025 Dec 12;158(10):2641–9. doi: 10.1002/ijc.70271 (PMC12996753; doi:10.1002/ijc.70271)
Supplement: Supplementary file 1 — DATA S1. Supporting Information. [file IJC-158-2641-s001.docx]

**Manuscript title:** Exercise serum promotes DNA damage repair and remodels gene expression in colon cancer cells

**Authors:** Samuel T Orange, Emily Dodd, Sharanya Nath, Hannah Bowden, Alastair Jordan, Hannah Smith, Ann Hedley, Ifeoma Chukwuma, Ian Hickson, Sweta Sharma Saha

| **Table of Contents** | **Page** |
| --- | --- |
| **Supplementary tables** |  |
| Table S1. Participant characteristics of the subsample (n = 12) used to generate pre- and post-exercise serum for RNA-seq analysis | 2 |
| Table S2. Sequencing coverage and quality statistics for each sample | 3-4 |
| Table S3. List of qRT-PCR primers | 5 |

| **Table S1. Participant characteristics of the subsample (n = 12) used to generate pre- and post-exercise serum for RNA-seq analysis** | |
| --- | --- |
|  | Total (n=12) |
| Age (years) | 60.1 ± 5.3 |
| Female | 5 (42%) |
| Ethnicity |  |
| White British | 11 (92%) |
| Multiple ethnic groups | 1 (8%) |
| V̇O_2_peak (ml.kg^-1^.min^-1^) | 27.5 ± 6.9 |
| Body mass (kg) | 84.2 ± 9.4 |
| BMI (kg/m^2^) | 29.4 ± 2.4 |
| Waist circumference (cm) | 96.2 ± 7.7 |
| Body fat (%) | 35.4 ± 7.3 |
| Fat-free mass (%) | 64.6 ± 7.1 |
| Skeletal muscle mass (%) | 35.4 ± 7.3 |
| Fasting BG (mmol/L) | 4.9 ± 0.7 |
| Peak expiratory flow (L/min) | 419 ± 122 |
| Data presented as mean ± SD or number (%)  BG = blood glucose; BMI = body mass index; V̇O_2_peak = peak oxygen consumption. | |

| **Table S2. Sequencing coverage and quality statistics for each sample** | | | | | | |
| --- | --- | --- | --- | --- | --- | --- |
| Sample ID | Total number of sequenced reads | Total number of uniquely mapped reads (GRCh38) | RNA integrity number (RIN) | Ratio of all reads aligned to rRNA regions to total uniquely mapped reads (rRNA rate) | Ratio of exon-mapped reads to total uniquely mapped reads (Expression Profile Efficiency) | Total number of detected transcripts with reads ≥1b |
| IR1 | 27779563 | 22654233.6 | 9.7 | 4.4142E-08 | 0.8155 | 21391 |
| IR2 | 27865258 | 22526274.6 | 9.7 | 1.3318E-07 | 0.8084 | 21021 |
| NU13Post | 43639984 | 30390884.9 | 9.7 | 1.3162E-07 | 0.6964 | 22169 |
| NU13PostIR | 37056532 | 31242362.1 | 9.8 | 0 | 0.8431 | 19988 |
| NU13Pre | 33399845 | 28847446.1 | 9.7 | 6.933E-08 | 0.8637 | 21932 |
| NU13PreIR | 35694759 | 31140107.8 | 9.7 | 9.6339E-08 | 0.8724 | 21768 |
| NU14Post | 31415326 | 23018009.4 | 9.7 | 4.3444E-08 | 0.7327 | 21812 |
| NU14PostIR | 29611559 | 25921958.7 | 9.8 | 1.1573E-07 | 0.8754 | 21804 |
| NU14Pre | 28874255 | 24979118 | 9.8 | 2.0017E-07 | 0.8651 | 21703 |
| NU14PreIR | 33411771 | 28684005.4 | 9.8 | 1.0459E-07 | 0.8585 | 21887 |
| NU17Post | 27314490 | 19838514.1 | 9.7 | 0 | 0.7263 | 21497 |
| NU17PostIR | 30778319 | 27118776.9 | 9.7 | 0 | 0.8811 | 21623 |
| NU17Pre | 29840726 | 26197173.4 | 9.8 | 7.6344E-08 | 0.8779 | 21785 |
| NU17PreIR | 31140231 | 27309982.6 | 9.8 | 7.3233E-08 | 0.877 | 21816 |
| NU18Post | 27135455 | 19760038.3 | 9.6 | 1.0121E-07 | 0.7282 | 21546 |
| NU18PostIR | 29861474 | 26095942.1 | 9.7 | 1.1496E-07 | 0.8739 | 21601 |
| NU18Pre | 32101732 | 27995920.5 | 9.7 | 7.1439E-08 | 0.8721 | 21755 |
| NU18PreIR | 29263989 | 25857660.7 | 9.7 | 1.5469E-07 | 0.8836 | 21781 |
| NU20Post | 31552801 | 21727258.8 | 9.6 | 9.205E-08 | 0.6886 | 21666 |
| NU20PostIR | 29313610 | 25280057.3 | 9.8 | 1.9778E-07 | 0.8624 | 21761 |
| NU20Pre | 27482545 | 24473206.3 | 9.8 | 1.2258E-07 | 0.8905 | 21592 |
| NU20PreIR | 27798917 | 23748614.8 | 9.7 | 4.2108E-08 | 0.8543 | 21566 |
| NU21Post | 31753964 | 21348190 | 9.8 | 0 | 0.6723 | 21724 |
| NU21PostIR | 26268092 | 22325251.4 | 9.8 | 8.9585E-08 | 0.8499 | 21669 |
| NU21PreIR | 35000700 | 30321106.4 | 9.8 | 9.8941E-08 | 0.8663 | 22006 |
| NU2Post | 27849658 | 21224224.4 | 9.7 | 4.7116E-08 | 0.7621 | 21691 |
| NU2PostIR | 32776719 | 28777959.3 | 9.8 | 6.9498E-08 | 0.878 | 21495 |
| NU2PreIR | 37185251 | 32812265.5 | 9.7 | 3.0476E-08 | 0.8824 | 20723 |
| NU3Post | 32420243 | 28325566.3 | 9.7 | 1.7652E-07 | 0.8737 | 21889 |
| NU3PostIR | 29363166 | 25983465.6 | 9.7 | 3.8486E-08 | 0.8849 | 21834 |
| NU3Pre | 28384409 | 24958410.8 | 9.1 | 4.0067E-08 | 0.8793 | 21519 |
| NU3PreIR | 30573368 | 26638575.5 | 9.8 | 1.877E-07 | 0.8713 | 21838 |
| NU6Post | 36525787 | 24264080.3 | 9.8 | 4.1213E-08 | 0.6643 | 21890 |
| NU6PostIR | 26753458 | 23403925.1 | 9.7 | 4.2728E-08 | 0.8748 | 21561 |
| NU6Pre | 34194140 | 30107940.3 | 9.8 | 1.3286E-07 | 0.8805 | 22103 |
| NU7Post | 35785068 | 23797070.2 | 9.5 | 0 | 0.665 | 21910 |
| NU7PostIR | 29180837 | 24762858.3 | 9.7 | 0 | 0.8486 | 21569 |
| NU7Pre | 34971451 | 31439334.4 | 9.8 | 3.1807E-08 | 0.899 | 22003 |
| NU7PreIR | 35439763 | 30552619.7 | 9.6 | 9.8191E-08 | 0.8621 | 21756 |
| NU8Post | 29637887 | 25740504.9 | 9.6 | 1.554E-07 | 0.8685 | 21899 |
| NU8PostIR | 32832713 | 28705641 | 9.7 | 6.9673E-08 | 0.8743 | 22108 |
| NU8Pre | 27571128 | 24127494.1 | 9.8 | 1.2434E-07 | 0.8751 | 21807 |
| NU8PreIR | 33141115 | 29701067.3 | 9.7 | 0 | 0.8962 | 21389 |
| NU9Post | 29426085 | 20409932.6 | 9.7 | 9.7992E-08 | 0.6936 | 21495 |
| NU9PostIR | 32513601 | 28134018.9 | 9.7 | 1.7772E-07 | 0.8653 | 21955 |
| NU9Pre | 32755950 | 27259501.6 | 9.1 | 1.1005E-07 | 0.8322 | 21901 |
| NU9PreIR | 26896060 | 24582998.8 | 9.5 | 8.1357E-08 | 0.914 | 21356 |
| UT1 | 33905051 | 30056827.7 | 9.7 | 3.327E-08 | 0.8865 | 21531 |
| UT2 | 40623044 | 35419232.1 | 9.7 | 1.1293E-07 | 0.8719 | 21745 |

| **Table S3. List of qRT-PCR primers** | |
| --- | --- |
| **Target Gene Name** | **Primer Sequence** |
| PNKP_F | TCATGTATGGCTACAGGAAG |
| PNKP_R | AGGAGAAACAGCGTTTATTG |
| PSME4_F | TAGCTGTTTGTTTAACAGC |
| PSME_R | GGGCTTCTTGTATCTTTCAC |
| NEIL_F | AGCAGTTCAGGGAGAATG |
| NEIl_R | CAGATAGTTGCCAATGCC |
| GAPDH_F | TCGGAGTCAACGGATTTG |
| GAPDH_R | CAACAATATCCACTTTACCAGAG |
